# Reduced oxidative stress suppresses neurotoxicity in the *Drosophila* model of TAF15-associated proteinopathies

**DOI:** 10.1186/s13041-022-00979-8

**Published:** 2022-11-21

**Authors:** Yeo Jeong Han, Kiyoung Kim

**Affiliations:** Department of Medical Science, Soonchunhyang University, 31538 Asan, Korea

**Keywords:** *Drosophila*, Oxidative stress, Proteinopathies, Glutathione transferase, TAF15, Neurodegenerative disease

## Abstract

TATA-binding protein associated factor 15 (TAF15) is involved in the pathology of amyotrophic lateral sclerosis (ALS) and frontotemporal dementia (FTD). Although TAF15 accumulates as cytoplasmic aggregates in neurons and the clearance of aggregates may be a therapeutic strategy for ALS, the underlying mechanisms of TAF15 in ALS remain poorly understood. Recently, we found that glutathione transferase omega 2 (GstO2) expression level is significantly reduced in the brain tissue of TAF15-expressing flies. In this study, we demonstrated that GstO2 overexpression in TAF15-induced flies rescues the locomotive activity and neuromuscular junctional defects. Furthermore, TAF15 levels in both cytoplasm and nuclear fractions significantly decreased in the heads of GstO2 co-expressing flies. GstO2-co-overexpression in neurons caused a marked decrease in intracellular reactive oxygen species generation in TAF15-induced flies. Our findings demonstrated that GstO2 was a pathogenic regulator of TAF15-associated proteinopathies. They help expand our understanding of TAF15-associated ALS pathogenesis.

**Supplementary Information:**

The online version contains supplementary material available at 10.1186/s13041-022-00979-8.

## Main text

Amyotrophic lateral sclerosis (ALS) is a fatal neurodegenerative disease characterized by a progressive loss of both the upper and lower motor neurons [1]. Mutations in TATA-binding protein associated factor 15 (*TAF15*) gene encoding an RNA-binding protein is implicated in the pathogenesis of familial and sporadic ALS, and ALS-linked variants of TAF15 form aggregates in the cytoplasm of neurons [2, 3]. As the clearance of cytoplasmic TAF15 aggregates could be a therapeutic strategy for ALS, identification of a TAF15 regulator for protection against TAF15-associated toxicity is essential. Recently, we found that glutathione transferase omega 2 (GstO2) expression level is significantly reduced in the brain tissue of TAF15-expressing flies [4]. Glutathione S‑transferases (GSTs) are multigene family phase II detoxifying enzymes that are activated during various stress conditions [5]. Using various model organisms, previous studies have demonstrated that the omega class GST (GSTO) is responsible for eliminating exogenous or endogenous stress [6–8]. Therefore, GSTOs may play critical roles in modulating cellular stress by regulating oxidative stress.

**Fig. 1:**
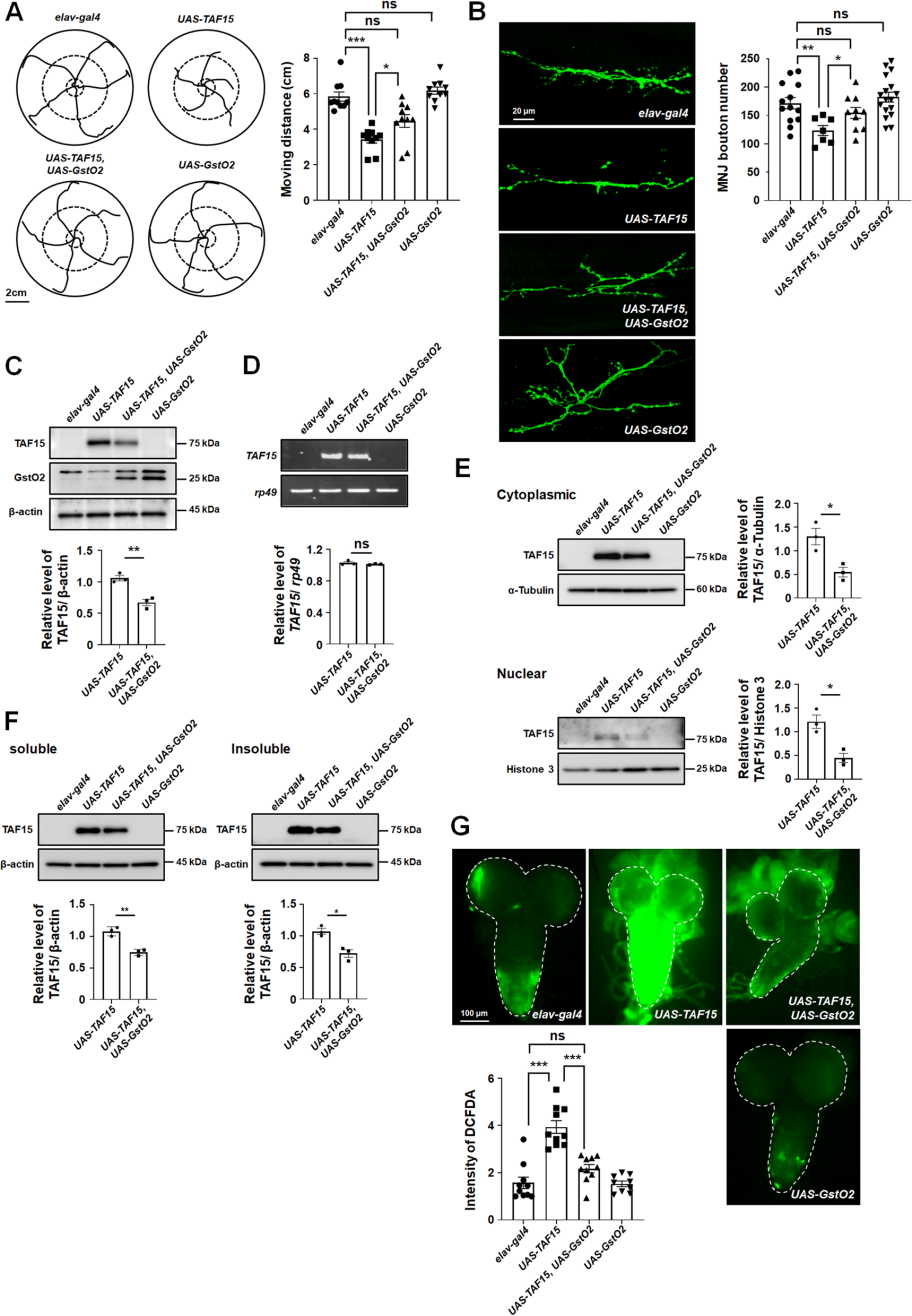
**A** GstO2 overexpression by *elav-Gal4*, a neuron-specific driver, partially restored the reduced locomotor activity in TAF15-overexpressing flies. The movement of third instar larvae on the agar plate was monitored for 90 s, and the average distance of the larval trails was calculated (n = 10 for each genotype). The error bars represent the mean ± standard deviation. Statistical significance was determined using one-way analysis of variance (ANOVA; *, *p* < 0.05; ***, *p* < 0.001; *ns* not significant). **B** Representative images of neuromuscular junction boutons labeled with anti-horseradish peroxidase at muscle 6/7 of segment 3 and quantification of the total number of boutons. TAF15 expression in the neurons of flies resulted in a significant reduction in the number of boutons. GstO2 expression in neuronal cells partially improved the reduced synaptic bouton number in TAF15-expressing flies. Error bars represent the mean ± standard deviation (*n* ≤ 7 for each genotype). Experimental significance was determined using one-way ANOVA (*p < 0.05; ***p < 0.001; *ns* not significant). **C** TAF15 levels in GstO2 co-expressing fly heads. TAF15 and GstO2 co-expression in neuronal cells resulted in a significant reduction in TAF15 levels. β-actin was used as a loading control. Error bars represent mean ± standard deviation of three independent experiments. Quantification of TAF15 levels was normalized to β‐actin (*n* = 3, independent preparations). Experimental significance was determined using Student’s *t* test (**, *p* < 0.01). **D** *TAF15* mRNA expression in the heads of TAF15-expressing flies. GstO2 expression did not affect *TAF15* mRNA levels. *rp49* was used as a loading control. Quantified *TAF15* mRNA levels were normalized to the *rp49* levels. Error bars represent the mean ± standard deviation of three independent experiments. The significance was determined using Student’s *t* test (*ns* not significant). **E** Both cytoplasmic and nuclear TAF15 levels were reduced by GstO2 overexpression in neuronal cells. Quantification of TAF15 levels normalized to α-tubulin (the cytoplasmic marker) and histone H3 (the nuclear marker) (*n* = 3, independent preparations). The error bars represent the standard deviation. Experimental significance was determined using Student’s *t* test (*, *p* < 0.05). **F** Soluble and insoluble fractions isolated from adult heads of the indicated genotypes were analyzed using immunoblotting. Both soluble and insoluble TAF15 levels were decreased by GstO2 overexpression. β-actin was used as a loading control. Quantification of TAF15 levels was normalized to β‐actin. The error bars represent the standard deviation of three independent experiments. Statistical significance was determined using Student’s *t* test (*, *p* < 0.05; **, *p* < 0.01). **G** 2′,7′-dichlorofluorescein (DCF) fluorescence (green) indicates reactive oxygen species (ROS). Increased ROS production in TAF15-expressing flies compared with that in the control flies. GstO2 co-expression suppressed ROS production in TAF15-expressing flies. Quantification of DCF fluorescence intensity in whole brains (dotted line) was performed using ImageJ software and normalized to the value of control flies. Experimental significance was determined using one-way ANOVA (***, *p* < 0.001; *ns* not significant; *n* ≤ 9 for each genotype)

In the present study, we aimed to examine the neuroprotective role of GSTO in the *Drosophila* model of TAF15-associated proteinopathies. We previously found that GstO2 expression suppressed TAF15-induced rough eye phenotype in fly models using modifier screening and GstO2 protein level was decreased in TAF15-expressing flies [4]. Given the strong evidence that GstO2 regulates TAF15-induced toxicity in flies, we determined the therapeutic potential and effect of GstO2 on TAF15-induced toxicity in the nervous system by testing the locomotor activity of the third instar larva. We generated flies expressing TAF15 and GstO2 in neurons by the pan-neuronal gal4 driver, elav-gal4 and quantified the crawling activity of the larvae by placing them on a grape juice agar plate for 90 s and measuring their crawling traces. Notably, the moving distance of TAF15-expressing larvae indicated a significant reduction compared with that of the control larvae. A shorter moving distance, induced by TAF15, was indicative of impaired locomotive activity in flies. Moreover, the distance crawled by larvae co-expressing TAF15 and GstO2 was longer than that of larvae expressing only TAF15 (Fig. 1A). Subsequently, we investigated the morphology of the neuromuscular junction (NMJ) in TAF15-expressing flies and measured the number of NMJ synaptic boutons by staining muscle 6/7 in segment A3 with anti-horseradish peroxidase-fluorescein-5-isothiocyanate. The NMJ synaptic boutons facilitated the presynaptic terminal transmission of motor neurons. The neuron-specific expression of TAF15 induced a 30% reduction in the number of synaptic boutons compared with the control flies (Fig. 1B). The defective NMJ synaptic bouton count is likely responsible for locomotor defects. Furthermore, GstO2 co-expression in neurons significantly reduced TAF15-induced synaptic bouton abnormalities (Fig. 1B), which led to the recovery of locomotor defects, as depicted in Fig. 1A. These results suggest that GstO2 and TAF15 genetically interact at the NMJ compartment to maintain the synaptic function of NMJs, and TAF15-induced neuronal toxicity can be significantly suppressed by increasing GstO2 expression.

Similar to other RNA-binding proteins, TAF15 is predominantly localized in the nucleus and forms stress granules in the cytoplasm under various pathological conditions [2, 9, 10]. Cytoplasmic TAF15 aggregates were found in the patients with ALS and FTD [2]. Therefore, the regulation of TAF15 stability in the cytoplasm is a critical therapeutic mechanism for TAF15-associated proteinopathies. First, we measured the expression level of TAF15 to determine its stability. Notably, the TAF15 levels were significantly decreased in the neurons of GstO2 co-expressing flies (Fig. 1C). Although the TAF15 levels were significantly decreased in the heads of GstO2 co-expressing flies, the amount of *TAF15* mRNA remained unaffected (Fig. 1D). These findings indicate that GstO2 regulates TAF15 stability. GstO2 suppressed TAF15 toxicity, which caused defects in the locomotive activity and NMJ synaptic boutons, by reducing the TAF15 level in neurons.

Next, to examine whether GstO2 expression regulates TAF15 mislocalization, we performed subcellular fractionation assays to measure TAF15 levels in both the cytoplasm and nucleus. Both cytoplasmic and nuclear TAF15 levels were significantly reduced in the neurons of GstO2-co-expressing flies (Fig. 1E). These results suggest that GstO2 expression mitigates the cytoplasmic TAF15 mislocalization. To investigate the effects of GstO2 on TAF15 aggregation, we separated the head extracts into detergent-soluble and -insoluble fractions. Notably, GstO2-co-expressing flies showed significantly decreased TAF15 levels in both soluble and insoluble fractions (Fig. 1F). These findings indicate that GstO2 expression alleviates TAF15-induced neuronal toxicity by reducing TAF15 aggregate formation in neurons.

Further, neuronal cells have increased oxidative stress in various neurodegenerative diseases, including ALS [11], and this is hypothesized to be a critical pathogenic mechanism of ALS. In addition, oxidative stress regulates the nuclear-cytoplasmic translocation of TDP-43 protein [12]. Therefore, mislocalized proteins in the cytoplasm, induced by reactive oxygen species (ROS), may contribute to cellular toxicity. To examine the protective mechanism of GstO2 on TAF15-induced toxicity, we measured intracellular ROS levels in the larval brain using the redox-sensitive fluorophore 2′,7′-dichlorofluorescein-diacetate (DCF-DA) staining. Notably, the ROS levels in neurons significantly increased in TAF15-expressing flies. Importantly, GstO2-co-overexpressing flies exhibited a marked decrease in intracellular ROS production (Fig. 1G). These results suggested that GstO2 reduced ROS production that was induced by TAF15 in the neurons of *Drosophila*.

This study presents the first evidence that GstO2 has a protective function in TAF15-associated neurodegenerative diseases. In conclusion, GstO2 is a target molecule for antioxidant defense mechanism against oxidative stress induced by TAF15. We demonstrated that GstO2 ameliorated the degenerative and defective phenotypes induced by TAF15 overexpression in neuronal cells. Notably, the ALS-linked TAF15 mutations, including M368T, G391E, R408C, and G473E, are more aggregation-prone in rat spinal cord neurons and have a more severe effect on lifespan than TAF15 wild-type when expressed in *Drosophila* [2]. Taken together, although further studies for the protective role of GstO2 on TAF15 variants-induced neurotoxicity in *Drosophila* are needed, these results indicate that GstO2 is important for the survival of neuronal cells in *Drosophila* model of TAF15-associated proteinopathies and required for the maintenance of locomotor function and neuromuscular junctional integrity in TAF15-induced disease condition. Therefore, our findings suggest that GstO2 can be used as a therapeutic mediator to treat neurodegenerative diseases caused by TAF15.

In addition, we recently reported that GstO2 significantly decreased fused in sarcoma (FUS) glutathionylation, a reversible post-translational modification, at Cys447 and reduced cytoplasmic FUS aggregate formation in neuronal cells of *Drosophila* [4]. Furthermore, TAF15 and FUS constitute the ten-eleven translocation family of RNA-binding proteins [13]. Similar to the FUS property both in vitro and in vivo, TAF15 forms aggregates in the cytoplasm, increasing neuronal toxicity. Therefore, we hypothesized that TAF15 also forms aggregates in the cytoplasm by inducing protein glutathionylation, and this glutathionylation regulated by GstO2 directly affects TAF15 neurotoxicity in neuronal cells of *Drosophila*. We will evaluate the effects of GstO2 on the regulation of TAF15 glutathionylation and TAF15-associated pathology in *Drosophila* in a future study. Moreover, our recent work showed that GstO2 suppresses oxidative damages, including excessive mitochondrial ROS production and increased protein oxidation, induced by FUS in *Drosophila* [4]. Therefore, GstO2 may play a protective role in the neuronal cells of *Drosophila* by suppressing the excessive ROS production from mitochondria in TAF15-associated proteinopathies. Clearly, further studies are necessary to better understand the molecular mechanisms by which expressing GstO2 protects against TAF15-associated neurodegeneration (Additional file 1).

## Supplementary Information

**Additional file 1. **Materials and methods.

## Data Availability

All data generated and/or analyzed during this study are included in this published article. Materials and methods are presented in the additional information.
