# Supplementary material for: Reduced oxidative stress suppresses neurotoxicity in the Drosophila model of TAF15-associated proteinopathies
Source: Mol Brain. 2022 Nov 21;15:93. doi: 10.1186/s13041-022-00979-8 (PMC9677661; doi:10.1186/s13041-022-00979-8)
Supplement: Supplementary file 1 — Additional file 1. Materials and methods. [file 13041_2022_979_MOESM1_ESM.doc]

**Reduced oxidative stress suppresses neurotoxicity in the *Drosophila* model of TAF15-associated proteinopathies**

Yeo Jeong Han and Kiyoung Kim^*^

Department of Medical Science, Soonchunhyang University, 31538 Asan, Korea

^*^Correspondence: Kiyoung Kim, Department of Medical Science, Soonchunhyang University, 31538 Asan, Korea; E-mail: kiyoung2[@sch.ac.kr](mailto:jyim@snu.ac.kr)

**Materials and methods**

***Drosophila stocks and genetics***

All stock flies were raised at 25 °C under normal humidity conditions (60%) and standard food. Crosses between flies were performed according to the standard procedure, and progeny was raised at a normal temperature or 29 °C. The UAS-TAF15 line was gifted by Nancy M. Bonini (University of Pennsylvania, Philadelphia, PA, USA). The UAS-GstO2 line has been described previously (1). The pan-neuronal driver, elav^3A^-Gal4, line was obtained from the Bloomington Drosophila Stock Center. In accordance with the genetic background, we used *W^1118^* flies as controls.

***Immunohistochemistry***

To analyze the neuromuscular junction (NMJ), third instar larvae were dissected in phosphate buffered saline (PBS) and fixed in 3% paraformaldehyde in PBS for 15 min. The samples were washed in PBS containing 0.1% Triton X-100 (PBST) for 10 min per wash and blocked with 5% BSA in PBST. Then, the samples were incubated with primary antibody for 12 h at 4°C. Fluorescein 5-isothiocyanate (FITC)-conjugated anti-horseradish peroxidase (HRP) (1:150; Jackson Immuno Research Laboratories, Cat#: 123-095-021) was used. Larval preparations were mounted in *SlowFadeTM* Gold antifade reagent (Invitrogen, Cat#: S36936). All images were collected using a Carl Zeiss confocal microscope (LSM710) at the Soonchunhyang Biomedical Research Core-Facility of Korea Basic Science Institute (KBSI).

***Locomotor activity***

For the larva crawling assay, third instar larvae were washed with PBS to remove the remaining food medium. Larvae were briefly dried on clean filter paper and placed on a 2% grape juice agar plate. The larvae of each genetic line were allowed to crawl for 90 s. To quantify the crawling distance of the larvae, we drew lines to track the larvae and measured the distance using ImageJ software. For averaging, at least 12 larvae from each transgenic line were used.

***Immunoblot assay***

Protein was extracted by homogenizing the heads of the flies using LDS sample buffer (Invitrogen, Cat#: NP0007). The total protein extracts (10 μg) were separated using a 4~12% gradient sodium dodecyl sulfate-polyacrylamide gel electrophoresis (SDS‐PAGE) and transferred to polyvinylidene fluoride membranes (Millipore, Cat#: IPVH00010). The membrane was blocked with 4% non-fat dry milk in tris-buffered saline (TBS) containing 0.1% Tween-20 (TBST) for 1 h and incubated with primary antibodies overnight. Primary antibodies, including rabbit anti-TAF15 (1:1000; GeneTex, Cat#: GTX77901), rabbit anti-*Drosophila* GstO2 (1:1000), rabbit anti-histone H3 (1:5000; Abcam, Cat#: ab1791), and rabbit anti-β-actin (1:5000; Cell Signaling Technology, Cat#: 4967L), were used. The membranes were washed using TBST and incubated in the following HRP-conjugated secondary antibody: goat anti-rabbit IgG HRP conjugate (1:2000; Millipore, Cat#: AP307P). Protein detection was performed using an ECL-Plus kit (Amersham, Cat#: RPN2106).

***Quantitative reverse transcription-polymerase chain reaction (qRT-PCR)***

Total RNA was extracted from fly heads with TRIzol reagent (Sigma-Aldrich, Cat#: T9424). cDNA was synthesized using Moloney murine leukemia virus reverse transcriptase (Promega, Cat#: M1701), and PCR was carried out for 25 cycles. The primers used were: *rp49*-For (GCT TCA AGA TGA CCA TCC GCC C), *rp49*-Rev (GGT GCG CTT GTT CGA TCC GTA AC), *TAF15*-For (TGA TGT GAG TAG GTA TGG AGA AG), and *TAF15*-Rev (TCA TGA ATT CAG GTC TTC TAG TG).

***Cellular fractionation assay***

For subcellular fractionation, the heads of 20 male flies were lysed in the reagent included in a nuclear extraction kit (Active Motif, Cat#: RPN2106) according to the manufacturer's protocol. The protein extracts in different subcellular fractions were mixed with SDS loading buffer, boiled, and subjected to SDS-PAGE for western blot analysis.

***Solubility assay***

The heads of twenty male flies were homogenized in lysis buffer without SDS (50 mm Tris-HCl, 150 mm NaCl, 5 mM EDTA, 0.1% NP-40, and 10% glycerol, pH 7.5). The homogenate samples were centrifuged at 100,000 ×*g* for 30 min at 4℃. The supernatant from this step was collected as the soluble fraction. The pellets were further extracted with 2× SDS loading buffer with 2% SDS, sonicated, and boiled for 10 min at 95 ℃. The supernatant from this step was used as the insoluble fraction.

***ROS measurement***

The third instar larvae were dissected on Sylgard plates using PBS buffer. The dissected larvae were incubated with 10 μM 2′,7′-dichlorofluorescin diacetate (DCF-DA) (Sigma-Aldrich, Cat#: D6883) for 30 min at 37 °C. The signals were observed using an MZ210F Leica fluorescence microscope (Wetzlar, Germany). Larval brains were outlined manually. The average pixel intensity of the signal in the whole brain area was measured and analyzed using the ImageJ software.

**References**

1. Kim K, Kim SH, Kim J, Kim H, Yim J. Glutathione s-transferase omega 1 activity is sufficient to suppress neurodegeneration in a Drosophila model of Parkinson disease. J Biol Chem. 2012;287(9):6628-41.
